# The Best of Two Different Visual Instructions in Improving Precision Ball-Throwing and Standing Long Jump Performances in Primary School Children

**DOI:** 10.3390/jfmk7010008

**Published:** 2022-01-10

**Authors:** Vincenzo Sorgente, Erez James Cohen, Riccardo Bravi, Diego Minciacchi

**Affiliations:** Department of Experimental and Clinical Medicine, Physiological Sciences Section, University of Florence, 50134 Florence, Italy; vincenzo.sorgente@unifi.it (V.S.); erezjames.cohen@unifi.it (E.J.C.)

**Keywords:** motor development, motor control, motor skills, physical education

## Abstract

Two observational learning approaches have been shown to be successful in improving children’s motor performances: one is “technique-focused”, another is “goal-focused”. In this study, we sought to compare the effectiveness of these two strategies, thus testing for the more efficient method of observational learning to enhance motor skills in primary school children. To this end, two experiments were designed. Experiment 1 involved a precision ball throwing task. Experiment 2 involved a standing long jump task. A total of 792 subjects (aged 6–11) participated in this study and were divided into technique-focus (Experiment 1 *n* = 200; Experiment 2 *n* = 66), goal-focus (Experiment 1 *n* = 195; Experiment 2 *n* = 68), and control groups (Experiment 1 *n* = 199; Experiment 2 *n* = 64). The experiments were divided into pretest, practice, and retention phases. During the practice phase, the technique-focus and goal-focus groups were given different visual instructions on how to perform the task. The results showed that children aged 10–11 belonging to the technique-focus group performed significantly better in the practice phase than both the goal-focus and the control group (*p* < 0.001), but only for the precision ball throwing task. These findings could be useful for training adaptation in the context of motor learning and skills acquisition.

## 1. Introduction

The use of visual cues and instructions has been shown to be beneficial for enhancing motor performances [1,2,3,4,5,6]. In particular, visual instructions serve an important role in motor learning, encouraging the acquisition of motor skills without the time-consuming process of trial-and-error learning [1,7]. Furthermore, visual instructions allow the learner to construct a mental representation of the desired performance, break the whole task into subcomponents, select essential information, and configurate appropriate strategies to reconstruct the task, ultimately leading to a more efficient performance [1,2,7,8,9]. In this regard, the process of learning a task by watching someone else performing this task is defined as observational learning [2]. Observing a model serves an important function in human motor development, as the learner benefits from the activation of a matching motor program by direct perceptual-motor mapping [1,7]. Visual instructions in the form of observational learning are generally found to be more effective than doing nothing [10] or than verbal instructions alone [11,12] in leading to optimal motor performance. However, during the process of motor learning, the amount of information provided to the learner should be carefully balanced. Indeed, too much information could negatively influence motor performances [13,14,15,16], especially in children, since they are considered to be novice performers on account of their lack of experience, unfamiliarity with skills, and low motor repertoire [17]. Consequently, in order to maximize each learning session, a fragmented approach of observational learning could prove to be useful, allowing the learner to focus specifically on a single aspect of the performance. In fact, it was shown that motor performances tend to improve when a few simple, easy-to-follow instructions are provided [3,5,6,10,18]. The question with a fragmented and reduced approach for motor skill acquisition is: In order to achieve better performances faster, which information is more relevant and thus more efficient?

To this end, there seems to be two principal lines of thought pertaining the optimization of the “learning by observing” process. The first is focusing the visual instructions on the final effector of the movement (i.e., a “goal-focused” visual instruction), whereas the second is focusing the visual instructions on the proper movement technique (i.e., a “technique-focused” visual instruction). For instance, several authors [7,19,20] stated that what the learner extracts from observing a model’s movement is the sole desired goal of the action, not the motor command or the kinematic primitives. Conversely, other research has shown that instructions that were related to the movement technique enhanced body cognition, motor performance, and motor learning compared to instructions that were not technique-related [6,21,22,23]. Instead, Krajenbrink et al. [24] and van der Loo et al. [2] argued that, ultimately, motor learning is allowed by the experience the subject undergoes during the process of repeated practice, regardless of the nature of the instructions. Though these approaches have their own merits, a direct comparison between the “technique-focus” and the “goal-focus” fragmented strategies of observational learning is still lacking. Moreover, despite the fact that typically developing children represent an important population for motor learning research, recent studies examining the effects of observational learning on motor performance have been predominantly conducted in children with autism spectrum disorder [25,26,27,28,29,30], using a wide variety of tasks including computer gaming [25], building a house with a set of bricks [26], peer-yoked contingency gaming [27], labeling pictures [28], and opening a box with a plastic stick [30].

Bearing in mind the significant role ascribed to adequate motor competence as a determinant of public health [31,32,33], social skills [34,35], cognitive skills [36,37], and academic achievement [38,39], a crucial goal for scholars, educators, and teachers should be to understand and promote the strategies that facilitate motor skills learning and performance during childhood [40,41]. Considering this past research, in the present study, we sought to determine the best of two observational learning methods to improve children’s motor performances. Specifically, we tested the more efficient approach between the technique-focused (i.e., how to prepare the task) and the goal-focused (i.e., how to execute the task) visual instructions in enhancing motor skills across late childhood during the execution of two complex motor tasks, such as the Precision Ball Throwing (PBT) and the Standing Long Jump (SLJ). In particular, the PBT has been defined as an object manipulation task [42], which requires mainly upper limbs and eye–hand coordination, whereas the SLJ has been defined as a locomotor task [42], which requires mainly lower limb coordination as well as strength/power elements. Since these two tasks represent different facets of motor performance, comparing the effectiveness of two observational learning strategies for different motor tasks allows a global overview in regard to efficient motor skills learning. Finally, previous studies have shown that from age 10 onwards, there is a major step-up in motor competence when assessing either fine motor skills, object manipulation skills, or locomotor skills [41,43,44,45,46]. This would be attributed to the fact that children of these ages experience a period of stabilization in physical growth and consolidation of both cognitive as well as neuromotor abilities [41,47,48]. Therefore, it would be plausible to expect a significant increase in motor performances for both the PBT and SLJ tasks from age 10 onwards.

## 2. Materials and Methods

### 2.1. Participants

The study was composed of two experiments, which are described in the specific sections below. Additionally, the minimum number of participants required for the experiments was determined by an a priori power analysis using G*Power, estimating a sample size of 165 subjects (f = 0.5, alpha at 0.05, with 95% power for ANOVA repeated measures within factors). Nevertheless, additional subjects were recruited in order to ensure a normal distribution of the data. Following Experiment 1, it was apparent that there was no additional benefit of a superfluous number of subjects. Therefore, we modulated the number of participants recruited for Experiment 2 accordingly.

A total of 792 subjects took part in the study. Specifically, 594 children participated in Experiment 1 (involving the PBT), while a total of 198 children participated in Experiment 2 (involving the SLJ) (Table 1). All of the participants were 6 to 11 years old and reported no known physical or intellectual deficits. None of the participants were involved in extracurricular sports practice in the last six months. The study protocol was approved by the Institutional Ethics Committee of Comitato Etico Area Vasta Centro AOUCareggi, Prot. N.0018234E, Rif. 63/12. Furthermore, all children provided assent and the parents/guardians provided informed consent. All of the participants were unfamiliar with the experimental tasks, and were all tested individually by the principal investigator and one research assistant, who was familiar with the purpose of the study.

### 2.2. Procedures

With regard to the PBT task (used in Experiment 1), several studies have assessed movement effectiveness by using outcome measures, such as accuracy in hitting a target throwing balls/darts (for a review, see Wulf [10]). Concerning the SLJ task (used in Experiment 2), this has been suggested as one of the most valid field-based methods to assess various facets of motor development, such as muscular strength and gross motor coordination [49,50,51]. Being part of a large number of international batteries, the SLJ has been suggested as a practical, time efficient, and cheap method of assessing muscular fitness in children [32,49,52]. Since the optimal execution of these two tasks requires different neuromotor abilities [14,31,53], this experimental design permits observation of the effect of visual instructions on motor learning from a multifaceted standpoint, rather than investigating a single aspect of motor learning (i.e., investigating only a precision task or a gross motor task).

The experiments were performed in a large, quiet room [22,54], where only the principal investigator and a research assistant were available [2]. There was no time limit for any of the conditions, as participants performed at their preferred speed [55]. For both experiments, each participant was randomly assigned to one of three groups, i.e., technique-focus, goal-focus, or control group (Table 2).

The technique-focus group was given visual instructions regarding how to efficiently prepare the execution of the task, whereas the goal-focus group was given visual instructions showing the final execution of the task. The control group did not receive any visual or verbal instruction throughout the experiment. Moreover, for each participant, the experiment spanned across two consecutive days, and consisted of three different phases, i.e., pretest, practice, and retention phases (Table 3).

Prior to the pretest phase, the principal investigator briefly explained the task to the subject. During the pretests, no specific verbal/visual instructions nor feedback were given. Shortly after the pretest phase, the practice phase was conducted. During the practice phase, two different pre-recorded video demonstrations of the motor skill were presented on an electronic tablet: one for the goal-focus group, and another for the technique-focus group. The model who appeared in the videos was skilled at performing the task [8]. We opted for a pre-recorded video demonstration in order to ensure consistency during administration [40]; previous research supports this method for visually demonstrating skills [40,56], as it was shown that children’s performance does not change when given a video demonstration in lieu of a live demonstration [40]. Since it has been suggested that children may be overwhelmed with different types of information [8], the videos contained no audio instructions. The control group did not watch any video demonstration, as participants were asked to perform the task with no other instructions provided. One day after the pretest and practice phases, the retention phase was conducted. During the retention phase, the participants were asked to perform the task again, as many times as in the practice phase. Moreover, in the retention phase, no further instructions or reminders were given for any of the groups.

### 2.3. Experiment 1: Precision Ball Throwing

The task was to throw polyethylene low density balls (1 cm in diameter) at a polypropylene circular target, which was 38 cm in diameter. The target had three concentric rings, each 3 cm in width, and a 3-cm-diameter bullseye in the center. The target’s surface was covered with felt, while each ball had Velcro (i.e., hook and loop strips) attached to its surface. Thanks to this setup, with each throw, the ball stuck to the target, allowing the subjects to observe the result of their shot. The bullseye’s height and distance were adjusted according to age [17,54]. Specifically, the target height for all groups was 1.22 m; the target distance for younger children (age 6–8) was 1.50 m, whereas the distance for older children (age 9–11) was 2.00 m [17,54]. To indicate the distances, two white tapes were placed at 1.50 m and 2.00 m from the target. The PBT task required participants to throw the balls aiming at the bullseye. The participants always threw with their dominant hand [17], which was determined by asking the participants to write their name on paper [57]. The participants’ PBT performances were scored in the following way: balls that hit the bullseye were given 3 points; balls that hit the innermost ring (i.e., the ring right outside of the bullseye) were given 2 points; and balls that hit the outermost ring were given 1 point. Balls that missed the target were given 0 points. Seeing that Emanuel et al. [54] reported that, in their study, the children were distracted by the balls they had already thrown and often asked to remove them from the target, in this study, the research assistant removed the ball after every single trial performed. The assistant made sure not to interfere with the visual field of the participants during the trials.

For the pretest phase, the participants performed five ball throws [21], for a possible maximum total score of 15. After the pretest, in the practice phase, the visual instruction stimulations were presented. For the technique-focus group, the video showed the optimal movement technique for performing the PBT task (i.e., feet and hand placement, stance, and ball grip), whereas for the goal-focus group, the video showed only the final movement execution of the PBT task (i.e., ball throw and arm follow-through). Each video lasted approximately 30 s [2]. The optimal technique regarding the PBT task was adapted from the research work of Kitsantas et al. [9] and van der Loo et al. [2], and is reported in Table 4. One day after the pretest and practice phases, the retention phase was conducted. On the retention tests, the target was placed at the same distance that was used during the practice [8]. For both the practice and retention phases, the participants completed seven blocks of three ball throws [58], for a possible maximum total score of 63.

### 2.4. Experiment 2: Standing Long Jump

The SLJ task required the participants to jump horizontally from a standing still position, moving their body as far as possible in a forward direction [32]. The performance in the SLJ was evaluated by the total jump distance, which is the horizontal distance from the take-off line to the mark made by the heel on landing, and was measured in centimeters with a tape measure [49]. The take-off line was evidenced by a tape on the floor. No steps backward or preparatory hops/runs were allowed.

After the pretest phase (consisting of two attempts), for the practice phase, the visual instruction stimulations were presented to both the technique-focus and goal-focus groups. For the technique-focus group, the video showed the optimal movement technique for performing the SLJ task (i.e., stance and take-off angle reaching), whereas for the goal-focus group, the video showed only the final movement execution of the SLJ task (i.e., the horizontal jump and landing). The control group participants did not watch any video demonstration. Each video lasted approximately 30 s [2]. The optimal technique regarding the SLJ performance was taken from Hraski et al. [50] and is reported in Table 5. One day after the pretest and practice phases, the retention phase was conducted. The participants performed two attempts of the SLJ for each phase of the experiment [49,52].

### 2.5. Data Collection

The data consisted of the scores that subjects were given for the experimental task (i.e., the PBT or the SLJ task). Specifically, each subject was given three scores, one for each experimental phase (i.e., pretest, practice, and retention). Regarding the PBT task, the performance score was obtained by summing all of the scores for the single trials, while regarding the SLJ task, the performances were scored by registering the longest jump distance.

### 2.6. Statistics

The data were analysed using IBM SPSS Statistics 26 software. Parametric analyses were conducted, as the Shapiro–Wilk test revealed a normal distribution of data (*p* = 0.000). First, in order to test whether the sample of subjects was homogeneous regarding motor performances, a two-way ANOVA was implemented to evaluate the differences in performance scores, both between ages (i.e., factor 1: 6, 7, 8, 9, 10, and 11 years of age) and between groups (i.e., factor 2: technique-focus, goal-focus, and control). Moreover, a mixed ANOVA was implemented to evaluate the differences in performance scores among two between-subjects factors (i.e., ages and groups) and one within-subjects factor (i.e., time, referring to the practice and retention phases). Both the two-way ANOVA and the mixed ANOVA were followed by the Bonferroni post hoc test for multiple comparisons.

## 3. Results

### 3.1. Experiment 1: Precision Ball Throwing

The results for the PBT scores are reported in Table 6.

**Figure 1:**
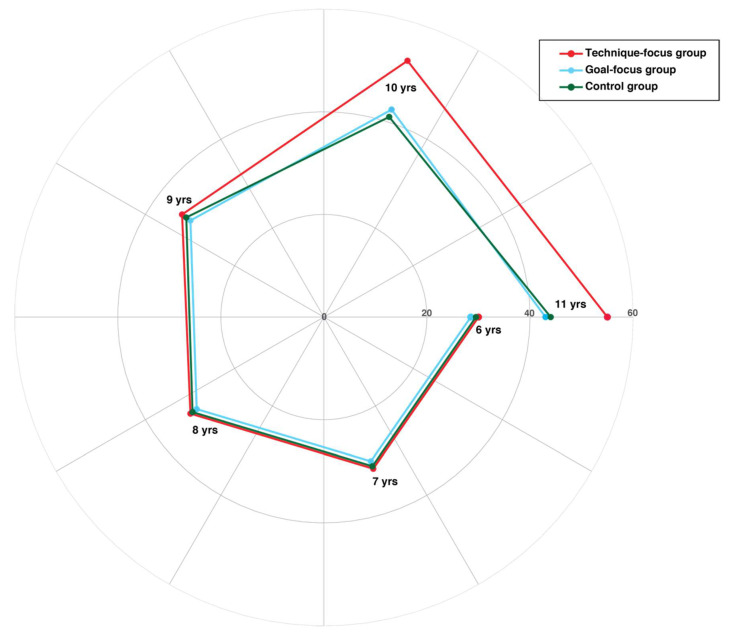
Polar diagram showing the mean scores obtained by all subjects for the PBT task during the practice phase. It is worth noting that while from age 6 to 9, the PBT scores were almost the same for all groups, subjects aged 10 and 11 of the technique-focus group (red line) performed the PBT task significantly better (*p* < 0.001) than both the goal-focus (cyan line) and control groups (green line).

For the pretest phase, the two-way ANOVA of the PBT performances for the factors group and age showed that there was no significant effect of experimental conditions on the PBT scores (F(10, 591) = 1.92, *p* = 0.14), meaning that the sample of subjects had the same starting level of motor skills. However, there was a significant effect of age on the PBT scores for all experimental conditions (F(5, 591) = 14.65, *p* < 0.001). Thus, as expected, the subjects’ motor performances tended to improve with age. In particular, the Bonferroni post hoc test for multiple comparisons showed a significant difference (*p* < 0.001) in PBT performances among all ages (6 vs. 7, 6 vs. 8, 6 vs. 9, and so forth). Furthermore, the mixed ANOVA showed that there was a significant interaction effect of age, experimental condition, and time on the PBT performances (F(10, 591) = 72.01, *p* = < 0.001). Specifically for the practice phase, we found that the mean score of the PBT for the technique group (mean = 39.16, standard deviation = 10.70) was significantly different (*p* < 0.001) than both the goal-focus (mean = 35.43, standard deviation = 4.38) and control groups (mean = 34.39, standard deviation = 5.83). This would denote that right after the visual instruction stimulation, the technique-focus group performed significantly better compared to the goal-focus and control groups. Interestingly, the Bonferroni post hoc test for multiple comparisons revealed that this significant difference in PBT scores among groups (*p* < 0.001) was present only for ages 10 and 11, whereas there were no group differences in PBT performances among the subjects aged 6–9 years (Figure 1).

However, for the retention phase, this effect was seemingly lost, as during this phase, the mean PBT performances for the technique-focus group (mean = 35.14, standard deviation = 6.08) did not significantly differ (*p* = 1.00) from the goal-focus (mean = 36.23, standard deviation = 5.10) and control groups (mean = 35.20, standard deviation = 5.98).

Moreover, the mean PBT score for the technique-group at the practice phase (mean = 39.16, standard deviation = 10.70) was significantly different (*p* < 0.001) from the mean PBT score of the same group at the retention phase (mean = 35.14, standard deviation = 6.08). This was not the case for the goal-focus and control groups, whose mean PBT scores did not significantly differ from the practice phase to the retention phase.

### 3.2. Experiment 2: Standing Long Jump

The descriptive statistics for the SLJ scores are reported in Table 7.

For the pretest phase, the two-way ANOVA of the SLJ performances for the factors group and age showed no significant effect of the experimental conditions on the SLJ scores (F(2, 195) = 1.82, *p* = 0.16), though there was an expected significant effect of age on the SLJ scores (F(5, 195) = 58.56, *p* < 0.001) among all experimental conditions. In particular, the Bonferroni post hoc test for multiple comparisons showed a significant difference (*p* < 0.001) in PBT performances among all ages (6 vs. 7, 6 vs. 8, 6 vs. 9, and so forth).

**Figure 2:**
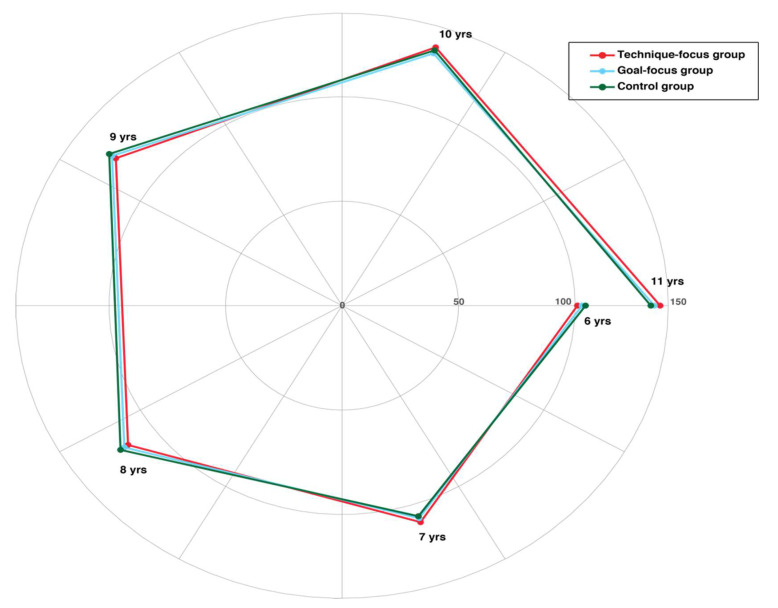
Polar diagram showing the mean scores obtained by all subjects for the SLJ task during the practice phase. Unlike the results from Experiment 1 using the PBT task, for Experiment 2, there was no significant difference in the SLJ performances (*p* = 0.24) among the technique-focus (red line), goal-focus (cyan line) and control (green line) groups.

Surprisingly, the mixed ANOVA returned no significant interaction effect of age, experimental condition, or time on the SLJ performances (F(10, 195) = 0.78, *p* = 0.65). Nonetheless, the test of the between-subjects effect showed that there was a significant effect of age on the SLJ performances (F(5, 195) = 891.86, *p* < 0.001), but this was not the case for the between-subjects factor or the within-subjects factor (F(2, 195) = 1.46, *p* = 0.24 and F(1, 195) = 0.17, *p* = 0.68, respectively). Hence, conversely to what we found for an object manipulation task like the PBT, for a locomotor task like the SLJ, there seems to be no performance enhancing effect of the two visual stimulations used in this experiment (Figure 2).

## 4. Discussion

The purpose of the present study, composed of two experiments, was to compare the effectiveness of two different strategies based on observational learning for improving motor performance in primary school children. One strategy was technique-focused, i.e., the subjects observed how to optimally prepare a motor task, with no further visual information regarding the ‘next steps’ of said movement. Conversely, the other strategy was goal-focused, i.e., the subjects observed the actual execution of the same motor task, with no previous visual information regarding the task ‘preparation’.

The results showed significant improvements of motor performance with age. Regarding Experiment 1, our findings showed that during the practice phase, the children of the technique-focus group aged 10–11 performed the PBT task significantly better than both the goal-focus and the control groups, whereas there were no significant differences among the experimental conditions for subjects aged 6 to 9 years old. Nevertheless, there was no retention of this apparent training effect brought by the technique-focused visual instruction. Concerning Experiment 2, we found no significant effect of experimental conditions on the SLJ performances, neither for the practice nor for the retention phases. Therefore, the results from this study are partially in line with previous investigations conducted on children with autism, which supported the use of observational learning strategies for enhancing motor performances [25,26,28,30]. Furthermore, our findings partly confirm that children aged 10–11 can efficiently use their short-term memory of action observation and can refine their performances without prior motor experience of certain complex motor tasks [1,11,26].

For both experiments, the motor performances significantly improved with age. This trend was expected, as competency in fundamental movement skills has been shown to follow an increasing developmental trajectory, with overall motor skills improving with chronological age [41,43,44,45,46,59,60].

Regarding the comparison of effectiveness between the two observational learning approaches, the results from Experiment 1 show that when it comes to efficiently enhancing children’s motor performance with visual instructions, a fragmented, technique-focused approach could be the most efficient. These outcomes are in line with previous research that has shown that instructions related to the movement technique enhanced motor performance in both children [22] and adults [21]. Seeing that these studies were conducted using verbal instructions, our study further elaborates on the topic of how to enhance children’s motor skills with the use of visual instructions. Interestingly, within the technique-focus group, only children aged 10–11 experienced a step-up in motor competence regarding the PBT performances. A reason for having found a significant training effect of the technique-focus condition on PBT performances may be due to more valuable information being extracted by the subjects from observing an efficient movement technique compared to the goal-focused approach, which focused solely on the movement execution. Seeing that a focus on movement technique effectively stimulates changes in posture for biomechanical efficiency, also allowing for the optimal use of physical capabilities including accuracy in a throwing task (for a review, see [61]), the movement pattern recreated by observing technique-focused visual instructions seems to be of significant use for improving the motor performances in children aged 10–11. In this regard, it is likely that subjects aged 10–11 may respond better to observational learning stimulations than their younger peers, based on the notion that children from age 10 onwards experience a period of stabilization in physical growth as well as maturation of both their cognitive and neuromotor capacities [41,47,48], thus being more capable of choosing primary information and configurating apt plans of action to reconstruct the task, eventually leading to better motor performances. However, another consequent takeaway of this finding is that other strategies should be pursued for improving motor skills in younger children, rather than a fragmented visual approach. Concerning this aspect, children aged 6–9 years old are considered to be quite far from having matured and efficient cognitive and neuromotor systems [17]; hence, they may need more information or different strategies for motor-enhancing purposes, e.g., involving verbal instructions, complete visual instructions, or a combination of the two, as well as providing visual/verbal feedback during motor performances.

Despite a partial training effect being found for the technique-focused condition on PBT performances for children aged 10–11 years old, the same effect was not maintained during the retention phase, as we observed two key findings. First, for the retention phase, there were no significant differences in PBT performance among the experimental conditions (i.e., technique-focus, goal-focus, control). Second, the retention performances of the technique-focus group were significantly less proficient compared to the performance scores obtained during the practice phase. Given that children are regarded as novice performers concerning motor skills acquisition [17], they may need an extended amount of practice in order to elicit any beneficial effects on their motor performance. Furthermore, retention was only tested in the short term, i.e., one day following the practice phase. Therefore, further studies are needed to learn more about the effect of observational learning strategies on long-term practice and the long-term retention of motor skills.

Moreover, while in Experiment 1 we found that a training effect was produced only by technique-focused visual instructions (though transient and only for subjects 10–11 years old), in Experiment 2, neither the technique-focused nor the goal-focused visual instructions produced a training effect on the subjects’ motor performances. First, it is worth mentioning that we took advantage of the “two-experiment” design to compare the improvements in motor performances between the two different facets of motor skills, i.e., object manipulation and locomotor skills. This was done in order to evaluate this experimental design from an overall standpoint in regard to motor skills. We certainly did not expect a complete ineffectiveness of the two observational learning strategies we shaped for improving the SLJ performances. Nonetheless, these contrasting results may be due to various reasons, such as the different neuromotor demands that the two tasks possess [14,31,53], as well as some prominent physical constraints due to the participants’ age. Specifically, while performing an object manipulation task (i.e., the PBT) mainly requires motor coordination of the dominant upper limb, performing a locomotor task (i.e., the SLJ) requires a completely different set of motor abilities, i.e., generating as much power as possible from mainly the lower limbs, along with coordinating the upper limbs and stabilizing the trunk. Hence, these neuromotor demands of strength and power may be too much of a physical constraint for primary school children, and consequently, there may be less room for technique-related approaches in non-developed individuals. Moreover, as Krajenbrink and colleagues already pointed out, one day of practice may be too short for training a complex gross motor task [24]. Therefore, as we already indicated for children 6–9 years old practicing the PBT task, more training time may be needed to induce and observe significant improvements for a locomotor task like the SLJ in primary school children. Finally, a viable option for future studies could be to measure hypothetical improvements in other aspects of locomotor skills performances rather than just the score of the task, e.g., biomechanical/kinematic parameters, perception of the motor performance, as well as including technique-related scores.

## 5. Conclusions

In response to the aim of this study, the more efficient approach of observational learning between the technique-focused and the goal-focused strategies seems to be the technique-focused one. However, in light of our results, this would be a partial and short-sighted response. In fact, other aspects are worth mentioning for future research to be carefully designed. Specifically, with this experimental design, a technique-focused strategy of observational learning can enhance children’s motor performances under specific conditions: if the children are at least 10–11 years old (not younger); right after the visual stimulation is provided (there seems to be no retention effect); and for a task that does not involve elements of strength/power, i.e., a precision task. Overall, the present study provides new insights into the best strategies for improving motor skills in primary school children. Moreover, our results have implications for all practical settings that involve motor performance and learning during childhood. Further studies are needed to shed more light on the influence that different approaches based on observational learning may have for improving motor proficiency during different stages of primary school education.

## Figures and Tables

**Table 1:** Number of participants per age and experiment.

Age (Years)	*n* (PBT ^1^)	*n* (SLJ ^2^)	*n* (PBT ^1^ + SLJ ^2^)
6	99	31	130
7	102	36	138
8	102	33	135
9	97	33	130
10	96	35	131
11	98	30	128
Total	594	198	792
M ^3^ ± SD ^4^	8.5 ± 1.87		

^1^ PBT = Precision Ball Throwing; ^2^ SLJ = Standing Long Jump; ^3^ M = mean; ^4^ SD = standard deviation.

**Table 2:** Number of subjects per age and experimental conditions.

	PBT ^1^			SLJ ^2^		
Age (Years)	T ^3^	G ^4^	C ^5^	T ^3^	G ^4^	C ^5^
6	32	33	34	9	12	10
7	36	34	32	12	11	13
8	35	33	34	10	13	10
9	34	30	33	14	10	9
10	30	31	35	10	13	12
11	33	34	31	11	9	10
Total	200	195	199	66	68	64

^1^ PBT = Precision Ball Throwing; ^2^ SLJ = Standing Long Jump; ^3^ T = technique-focus group; ^4^ G = goal-focus group; ^5^ C = control group.

**Table 3:** Timeline of the experiment.

Experimental Phase	Pretest	Practice	Retention
Activity	Baseline evaluation of the task	Visual instruction stimulation before performing the task	Re-perform the task with no further visual instructions
Day #	Day 1	Day 1	Day 2
Number of trials	PBT ^1^ = 5 SLJ ^2^ = 2	PBT ^1^ = 7 × 3 ^3^ SLJ ^2^ = 2	PBT ^1^ = 7 × 3 ^3^ SLJ ^2^ = 2

^1^ PBT = Precision Ball Throwing; ^2^ SLJ = Standing Long Jump; ^3^ 7 × 3 = seven blocks of three ball-throws.

**Table 4:** Based on the group (technique-focus or goal-focus), different subcomponents of the PBT performance were shown in the video demonstrations.

Technique-Focus Group	Goal-Focus Group
Grip: Holding the ball between the first and second finger and the thumb	Throw: Only the forearm and wrist are used to throw the ball
Stance: The right foot is slightly ahead of the left foot	Follow-through: After releasing the ball, allow the arm to continue its natural motion

**Table 5:** Based on the group (technique-focus or goal-focus), different subcomponents of the SLJ performance were shown in the video demonstrations.

Technique-Focus Group	Goal-Focus Group
Stance: The feet are shoulder-width apart	Horizontal jump: Jump as far as possible while extending the arms, hips, and legs
Take-off angle reaching: Squat while forward shifting the bodyweight, bending the knees at 90 degrees. While squatting, the arms go forward	Landing: the arms sweep forward and down to the hips. The feet are extended out until hitting the ground. The knees and hips absorb the impact as the body continues to move forward

**Table 6:** PBT performance scores ^1^ per age, group, and experimental phase.

	T ^2^			G ^3^			C ^4^		
Age	Pre ^5^	Pract. ^6^	Ret. ^7^	Pre ^5^	Pract. ^6^	Ret. ^7^	Pre ^5^	Pract. ^6^	Ret. ^7^
6	9.5 (1)	30 (2)	29 (2.25)	9 (2.25)	29 (1)	29 (2.75)	8 (2)	30 (1.25)	28 (2)
7	10 (1.25)	31 (2.50)	30 (2)	9 (3)	31 (1.75)	30 (2.75)	9 (2)	31 (2)	29 (3)
8	10 (3)	32 (2)	32 (3.25)	9.5 (3)	32 (1)	32 (2)	10 (3)	32 (3)	32 (2)
9	10.5 (2)	34 (2.75)	33 (3)	10 (3.25)	33 (3)	34 (3)	9.5 (3)	33 (2)	34 (3)
10	10 (2)	52 (3)	42 (1)	10 (2)	42.5 (2.75)	42 (2.25)	10 (2.75)	42 (3)	43 (3)
11	12 (3)	55 (4)	43 (3)	11 (3)	44 (2)	44 (3)	11 (3)	44 (3.5)	43 (3)

^1^ Median (interquartile range); ^2^ T = technique-focus group; ^3^ G = goal-focus group; ^4^ C = control group; ^5^ Pre = pretest phase; ^6^ Pract. = practice phase; ^7^ Ret. = retention phase.

**Table 7:** SLJ performance scores ^1^ per age, group, and experimental phase.

	T ^2^			G ^3^			C ^4^		
Age	Pre ^5^	Pract. ^6^	Ret. ^7^	Pre ^5^	Pract. ^6^	Ret. ^7^	Pre ^5^	Pract. ^6^	Ret. ^7^
6	103.2 (3)	103.1 (2.55)	102.9 (3.60)	103.2 (3.75)	103.8 (2.50)	102.9 (2.90)	102.9 (2.25)	102.8 (3.18)	103.2 (3.75)
7	108.5 (4.21)	107.3 (4)	109.3 (5.60)	107.2 (4.08)	107.9 (3.81)	110.8 (2.61)	109.6 (3.20)	108.4 (4.22)	108.5 (4.20)
8	115.8 (5.50)	118.7 (3.75)	119 (7.20)	119 (5.45)	114.9 (4.15)	116 (3.40)	117.5 (3.60)	114.5 (7.70)	117.2 (7.80)
9	121.6 (7)	121.5 (4.1)	123 (8)	123.4 (5.32)	122.9 (10.22)	122.47 (6.46)	122.3 (3)	122 (8.5)	122.5 (7)
10	130 (4.70)	129.3 (5.62)	124 (6.34)	130.1 (7.97)	127 (5.50)	126.5 (8.81)	125 (7.47)	130.1 (8.34)	129.8 (4.56)
11	138 (3.81)	136.2 (3.55)	134.8 (2.76)	134 (2.17)	136 (4.92)	136.45 (5.15)	136.5 (4.06)	135.6 (3.37)	136.2 (3.95)

^1^ Median (interquartile range); ^2^ T = technique-focus group; ^3^ G = goal-focus group; ^4^ C = control group; ^5^ Pre = pretest phase; ^6^ Pract. = practice phase; ^7^ Ret. = retention phase.

## Data Availability

Data are available on request.

## References

[1] Hebert E. (2018). The Effects of Observing a Learning Model (or Two) on Motor Skill Acquisition. J. Mot. Learn. Dev..

[2] van der Loo J., Krahmer E., van Amelsvoort M. (2021). Learning How to Throw Darts. Effects of Modeling Type and Reflection on Novices’ Dart-Throwing Skills. J. Mot. Behav..

[3] Karlin L., Mortimer R.G. (1962). Effects of Visual and Verbal Cues on Learning a Motor Skill. J. Exp. Psychol..

[4] Bläsing B.E., Coogan J., Biondi J., Schack T. (2018). Watching or Listening: How Visual and Verbal Information Contribute to Learning a Complex Dance Phrase. Front. Psychol..

[5] Maraj B.K.V., Li L., Hillman R., Jeansonne J.J., Ringenbach S.D. (2003). Verbal and Visual Instruction in Motor Skill Acquisition for Persons with and Without Down Syndrome. Adapt. Phys. Act. Q..

[6] Hodges N., Franks I. (2004). Instructions, Demonstrations and the Learning Process. Skill Acquisition in Sport.

[7] Bekkering H., Wohlschläger A., Gattis M. (2000). Imitation of Gestures in Children Is Goal-Directed. Q. J. Exp. Psychol. Sect. A.

[8] Wulf G., Chiviacowsky S., Schiller E., Ávila L.T.G. (2010). Frequent External-Focus Feedback Enhances Motor Learning. Front. Psychol..

[9] Kitsantas A., Zimmerman B.J., Cleary T. (2000). The Role of Observation and Emulation in the Development of Athletic Self-Regulation. J. Educ. Psychol..

[10] Wulf G., Shea C., Lewthwaite R. (2010). Motor Skill Learning and Performance: A Review of Influential Factors. Med. Educ..

[11] de Stefani E., Rodà F., Volta E., Pincolini V., Farnese A., Rossetti S., Pedretti F., Ferrari P.F. (2020). Learning New Sport Actions: Pilot Study to Investigate the Imitative and the Verbal Instructive Teaching Methods in Motor Education. PLoS ONE.

[12] Williams A.M., Ford P.R. (2008). Expertise and Expert Performance in Sport. Int. Rev. Sport Exerc. Psychol..

[13] Lee T.D., Eliasz K.L., Gonzalez D., Alguire K., Ding K., Dhaliwal C. (2016). On the Role of Error in Motor Learning. J. Mot. Behav..

[14] Guadagnoli M.A., Lee T.D. (2004). Challenge Point: A Framework for Conceptualizing the Effects of Various Practice Conditions in Motor Learning. J. Mot. Behav..

[15] Roller M.L., Lazaro R.T., Byl N.N., Umphred D.A. (2013). Contemporary Issues and Theories of Motor Control, Motor Learning, and Neuroplasticity. Neurol. Rehabil..

[16] Schmidt R., Lee T., Winstein C., Wulf G., Zelaznik H. (2019). Motor Control and Learning: A Behavioral Emphasis, 6th Edition. Med. Sci. Sports Exerc..

[17] Fathi Khatab S., Ghasemi A., Mousavi Sadati S.K. (2018). The Effect of Focus Instructions on Dart Throwing Performance in Children With and Without Developmental Coordination Disorder. Ann. Appl. Sport Sci..

[18] Popp N.J., Yokoi A., Gribble P.L., Diedrichsen J. (2020). The Effect of Instruction on Motor Skill Learning. J. Neurophysiol..

[19] Hayes S.J., Ashford D., Bennett S.J. (2008). Goal-Directed Imitation: The Means to an End. Acta Psychol..

[20] Catmur C., Heyes C. (2019). Mirroring ‘Meaningful’ Actions: Sensorimotor Learning Modulates Imitation of Goal-Directed Actions. Q. J. Exp. Psychol..

[21] Wulf G., McNevin N.H., Fuchs T., Ritter F., Toole T. (2000). Attentional Focus in Complex Skill Learning. Res. Q. Exerc. Sport.

[22] Agar C., Humphries C.A., Naquin M., Hebert E., Wood R. (2016). Does Varying Attentional Focus Affect Skill Acquisition in Children? A Comparison of Internal and External Focus Instructions and Feedback. Phys. Educ..

[23] Benjaminse A., Welling W., Otten B., Gokeler A. (2018). Transfer of Improved Movement Technique after Receiving Verbal External Focus and Video Instruction. Knee Surg. Sports Traumatol. Arthrosc..

[24] Krajenbrink H., van Abswoude F., Vermeulen S., van Cappellen S., Steenbergen B. (2018). Motor Learning and Movement Automatization in Typically Developing Children: The Role of Instructions with an External or Internal Focus of Attention. Hum. Mov. Sci..

[25] MacDonald J., Ahearn W.H. (2015). Teaching Observational Learning to Children with Autism. J. Appl. Behav. Anal..

[26] Foti F., Piras F., Vicari S., Mandolesi L., Petrosini L., Menghini D. (2019). Observational Learning in Low-Functioning Children With Autism Spectrum Disorders: A Behavioral and Neuroimaging Study. Front. Psychol..

[27] Luke N., Singh N. (2018). Teaching Observational Learning to Children with Autism: Pedagogical Advancements for the Scientist-Practitioner. Int. J. Pedagog. Teach. Educ..

[28] DeQuinzio J.A., Taylor B.A., Tomasi B.J. (2018). Observational Learning and Children with Autism: Discrimination Training of Known and Unknown Stimuli. J. Appl. Behav. Anal..

[29] Taylor B.A., DeQuinzio J.A. (2012). Observational Learning and Children With Autism. Behav. Modif..

[30] Nadel J., Aouka N., Coulon N., Gras-Vincendon A., Canet P., Fagard J., Bursztejn C. (2011). Yes They Can!. Autism.

[31] Lloyd M., Saunders T.J., Bremer E., Tremblay M.S. (2014). Long-Term Importance of Fundamental Motor Skills: A 20-Year Follow-Up Study. Adapt. Phys. Act. Q..

[32] Sgrò F., Mango P., Pignato S., Schembri R., Licari D., Lipoma M. (2017). Assessing Standing Long Jump Developmental Levels Using an Inertial Measurement Unit. Percept. Mot. Ski..

[33] Sgrò F., Quinto A., Messana L., Pignato S., Lipoma M. (2017). Assessment of Gross Motor Developmental Level in Italian Primary School Children. J. Phys. Educ. Sport.

[34] Ohara R., Kanejima Y., Kitamura M., Izawa K.P. (2019). Association between Social Skills and Motor Skills in Individuals with Autism Spectrum Disorder: A Systematic Review. Eur. J. Investig. Health Psychol. Educ..

[35] Holloway J.M., Long T.M. (2019). The Interdependence of Motor and Social Skill Development: Influence on Participation. Phys. Ther..

[36] Herold F., Hamacher D., Schega L., Müller N.G. (2018). Thinking While Moving or Moving While Thinking—Concepts of Motor-Cognitive Training for Cognitive Performance Enhancement. Front. Aging Neurosci..

[37] Veldman S.L.C., Santos R., Jones R.A., Sousa-Sá E., Okely A.D. (2019). Associations between Gross Motor Skills and Cognitive Development in Toddlers. Early Hum. Dev..

[38] de Bruijn A.G.M., Kostons D.D.N.M., van der Fels I.M.J., Visscher C., Oosterlaan J., Hartman E., Bosker R.J. (2019). Importance of Aerobic Fitness and Fundamental Motor Skills for Academic Achievement. Psychol. Sport Exerc..

[39] Escolano-Pérez E., Herrero-Nivela M.L., Losada J.L. (2020). Association Between Preschoolers’ Specific Fine (But Not Gross) Motor Skills and Later Academic Competencies: Educational Implications. Front. Psychol..

[40] Palmer K.K., Matsuyama A.L., Irwin J.M., Porter J.M., Robinson L.E. (2017). The Effect of Attentional Focus Cues on Object Control Performance in Elementary Children. Phys. Educ. Sport Pedagog..

[41] Sorgente V., Cohen E.J., Bravi R., Minciacchi D. (2021). Crosstalk between Gross and Fine Motor Domains during Late Childhood: The Influence of Gross Motor Training on Fine Motor Performances in Primary School Children. Int. J. Environ. Res. Public Health.

[42] Ulrich D.A. (2017). Introduction to the Special Section: Evaluation of the Psychometric Properties of the TGMD-3. J. Mot. Learn. Dev..

[43] Cohen E.J., Bravi R., Bagni M.A., Minciacchi D. (2018). Precision in Drawing and Tracing Tasks: Different Measures for Different Aspects of Fine Motor Control. Hum. Mov. Sci..

[44] McKenzie T.L., Sallis J.F., Broyles S.L., Zive M.M., Nader P.R., Berry C.C., Brennan J.J. (2002). Childhood Movement Skills: Predictors of Physical Activity in Anglo American and Mexican American Adolescents?. Res. Q. Exerc. Sport.

[45] Okely A.D., Booth M.L., Chey T. (2004). Relationships between Body Composition and Fundamental Movement Skills among Children and Adolescents. Res. Q. Exerc. Sport.

[46] Bolger L.E., Bolger L.A., O’ Neill C., Coughlan E., O’Brien W., Lacey S., Burns C. (2018). Age and Sex Differences in Fundamental Movement Skills Among a Cohort of Irish School Children. J. Mot. Learn. Dev..

[47] Cohen E.J., Bravi R., Minciacchi D. (2021). Assessing the Development of Fine Motor Control in Elementary School Children Using Drawing and Tracing Tasks. Percept. Mot. Ski..

[48] Albuquerque M.R., Rennó G.V.C., Bruzi A.T., Fortes L.D.S., Malloy-Diniz L.F. (2021). Association between Motor Competence and Executive Functions in Children. Appl. Neuropsychol. Child..

[49] Gontarev S., Zivkovic V., Velickovska L.A., Naumovski M. (2014). First Normative Reference of Standing Long Jump Indicates Gender Difference in Lower Muscular Strength of Macedonian School Children. Health.

[50] Hraski M., Hraski Ž., Prskalo I. (2015). Comparison of Standing Long Jump Technique Performed by Subjects from Different Age Groups. Balt. J. Sport Health Sci..

[51] Sidhu J.S. (2018). Physical Attributes as Indicator of Performance for Broad Jumping. Int. J. Curr. Res. Rev..

[52] Wu W.-L., Wu J.-H., Lin H.-T., Wang G.-J. (2003). Biomechanical Analysis of the Standing Long Jump. Biomed. Eng. Appl. Basis Commun..

[53] Haywood Kathleen M., Getchell N. (2005). Life Span. Motor Development.

[54] Emanuel M., Jarus T., Bart O. (2008). Effect of Focus of Attention and Age on Motor Acquisition, Retention, and Transfer: A Randomized Trial. Phys. Ther..

[55] Southard D. (2011). Attentional Focus and Control Parameter. Res. Q. Exerc. Sport.

[56] Olivier I., Palluel E., Nougier V. (2008). Effects of Attentional Focus on Postural Sway in Children and Adults. Exp. Brain Res..

[57] Jongbloed-Pereboom M., Nijhuis-van der Sanden M.W.G., Steenbergen B. (2013). Norm Scores of the Box and Block Test for Children Ages 3–10 Years. Am. J. Occup. Ther..

[58] Lohse K.R., Sherwood D.E., Healy A.F. (2010). How Changing the Focus of Attention Affects Performance, Kinematics, and Electromyography in Dart Throwing. Hum. Mov. Sci..

[59] Williams H.G., Pfeiffer K.A., O’Neill J.R., Dowda M., McIver K.L., Brown W.H., Pate R.R. (2008). Motor Skill Performance and Physical Activity in Preschool Children. Obesity.

[60] Bravi R., Ioannou C.I., Minciacchi D., Altenmüller E. (2019). Assessment of the Effects of Kinesiotaping on Musical Motor Performance in Musicians Suffering from Focal Hand Dystonia: A Pilot Study. Clin. Rehabil..

[61] Holfelder B., Schott N. (2014). Relationship of Fundamental Movement Skills and Physical Activity in Children and Adolescents: A Systematic Review. Psychol. Sport Exerc..

